# Evaluation of Bioactive Properties of Lipophilic Fractions of Edible and Non-Edible Parts of *Nasturtium officinale* (Watercress) in a Model of Human Malignant Melanoma Cells

**DOI:** 10.3390/ph15020141

**Published:** 2022-01-25

**Authors:** Sotiris Kyriakou, Venetia Tragkola, Heba Alghol, Ioannis Anestopoulos, Tom Amery, Kyle Stewart, Paul G. Winyard, Dimitrios T. Trafalis, Rodrigo Franco, Aglaia Pappa, Mihalis I. Panayiotidis

**Affiliations:** 1 Department of Cancer Genetics, Therapeutics & Ultrastructural Pathology, The Cyprus Institute of Neurology & Genetics, Nicosia 2371, Cyprus; sotirisk@cing.ac.cy (S.K.); venetiat@cing.ac.cy (V.T.); ioannisa@cing.ac.cy (I.A.); 2 The Cyprus School of Molecular Medicine, The Cyprus Institute of Neurology & Genetics, Nicosia 2371, Cyprus; algholh@cing.ac.cy; 3 The Watercress Company, Dorchester DT2 8QY, UK; tom.amery@thewatercresscompany.com; 4 Watercress Research Limited, Devon TQ12 4AA, UK; kyle@watercress-research.com (K.S.); paul@watercress-research.com (P.G.W.); 5 Laboratory of Pharmacology, Medical School, National & Kapodistrian University of Athens, 11527 Athens, Greece; dtrafal@med.uoa.gr; 6 Redox Biology Centre, University of Nebraska-Lincoln, Lincoln, NE 68583, USA; rodrigo.franco@unl.edu; 7 Department of Veterinary Medicine & Biomedical Sciences, University of Nebraska-Lincoln, Lincoln, NE 68583, USA; 8 Department of Molecular Biology & Genetics, Democritus University of Thrace, 68100 Alexandroupolis, Greece; apappa@mbg.duth.gr

**Keywords:** watercress, phenethyl isothiocyanate, flavonoids, phenols, pigments, antioxidants, malignant melanoma, reactive oxygen species

## Abstract

Watercress is an enriched source of phenethyl isothiocyanate (PEITC), among other phytochemicals, with an antioxidant capacity. The aim of this study was to (i) chemically characterize and (*ii*) biologically evaluate the profile of the main health-promoting compounds contained in edible (i.e., mixture of leaves and lateral buds) and non-edible (i.e., stems) parts of watercress in an in vitro model of malignant melanoma consisting of human malignant melanoma (A375), non-melanoma (A431) and keratinocyte (HaCaT) cells. The extraction of the main constituents of watercress was performed by subjecting the freeze-dried edible and non-edible samples through different extraction protocols, whereas their concentration was obtained utilizing analytical methodologies. In addition, cell viability was evaluated by the Alamar Blue assay, whereas levels of oxidative stress and apoptosis were determined by commercially available kits. The edible watercress sample contained a higher amount of various nutrients and phytochemicals in the hexane fraction compared to the non-edible one, as evidenced by the presence of PEITC, phenolics, flavonoids, pigments, ascorbic acid, etc. The cytotoxicity potential of the edible watercress sample in the hexane fraction was considerably higher than the non-edible one in A375 cells, whereas A431 and HaCaT cells appeared to be either more resistant or minimally affected, respectively. Finally, levels of oxidative stress and apoptotic induction were increased in both watercress samples, but the magnitude of the induction was much higher in the edible than the non-edible watercress samples. Herein, we provide further evidence documenting the potential development of watercress extracts (including watercress waste by-products) as promising anti-cancer agent(s) against malignant melanoma cells.

## 1. Introduction

Watercress (*Nasturtium officinale*) is a cruciferous perennial plant of the *Brassicaceae* family, which includes broccoli, cauliflower, cabbage, horseradish, wasabi, etc. Like other *Brassicaceae* vegetables, watercress is a rich source of glucosinolates (GLs), a class of nitrogenous, sulfur-enriched phytochemicals that are linked to *D*-glucose molecules via a *β*-*D*-thioglucosidic bond [1,2]. GLs are stable and biologically inactive phytochemicals. However, upon plant tissue damage, they can be rapidly rearranged into biologically active secondary metabolites, including isothiocyanates (ITCs), nitriles, thiocyanates, epithionitriles and oxazolidine-2-thiones, depending on the substrate, pH and availability of ferrous ions [3,4,5]. Under physiological pH, ITCs are the major metabolic products [4,5]. The degradation of GLs is highly regulated by the action of an endogenous myrosinase (*β*-*D*-thioglucosideglucohydrolase), or by a myrosinase found in human gut bacteria, upon ingestion [6,7]. The major GLs produced by *Brassicacea* vegetables are methylsulfinylalkyl (e.g., glucoiberin, glucoraphanin and glycoalyssin) [8,9], olefinic (e.g., gluconapin, progoitrin and sinigrin) [10] and aromatic (e.g., gluconasturtiin) [11].

It is well documented, in various epidemiological studies, that there is a strong positive correlation between the daily supplementation of watercress and reduced risks of cardiovascular disease, diabetes and cancer [12,13]. Watercress comprises the richest known natural source of gluconasturtiin; the precursor of phenethyl ITC (PEITC), consisting approximately 94% of watercress content and whose biological action has been extensively studied in both in vivo and in vitro cancer models [14,15]. Specifically, it has been reported that PEITC has the capacity to act as a chemopreventing agent against a broad spectrum of cancers, including prostate, leukemia, cervical, liver, colon, lung, multiple myeloma and breast [16,17,18,19,20,21,22]. In addition, numerous reports suggested that PEITC promotes the activation of apoptotic cascades, thus making it a candidate anti-cancer drug, and, as such, it is currently undergoing phase I and II clinical trials [23,24,25]. Another key characteristic of the *Brassicaceae* vegetables is their recognition as a source of powerful antioxidants, including polyphenols, flavonoids, terpenoids and vitamins, all of which can act synergistically and/or additively. Boyd et al. (2006) have shown, for the first time, that a watercress extract can inhibit the proliferation and metastasis of HT-29 colon cancer cells by suppressing the progression of oxidative DNA damage [26,27]. In another study, the invasion and metastatic potential of human breast cancer (MDA-MB-23) cells was inhibited after treatment with the watercress extract [28]. This effect was attributed to the high content of reducing agents, metal chelators and lipid peroxidation inhibitors within the watercress extract, thereby making it a powerful scavenger of reactive oxygen species (ROS) [29,30,31].

In the current study, we have determined the chemical composition of the major nutrients (soluble sugars, proteins and ascorbic acid), as well as the composition of various bioactive phytochemicals (polyphenols, flavonoids, pigments and PEITC) in both edible and non-edible parts of watercress. Furthermore, we have evaluated the biological potency of watercress extracts in an in vitro model of human malignant melanoma consisting of primary malignant melanoma (A375), non-melanoma epidermoid skin carcinoma (A431) and non-tumorigenic immortalized keratinocyte (HaCaT) cells. Given the unmet clinical need for more efficient therapeutics in treating malignant melanoma (together with the observed cytotoxicity-induced side effects of current therapeutic regimens), naturally occurring compounds may offer an alternative therapeutic strategy, either as a monotherapy or in combination with other clinically relevant therapeutic protocols.

## 2. Results

### 2.1. Extraction of PEITC from Edible and Non-Edible Watercress Samples

**Figure 1:**
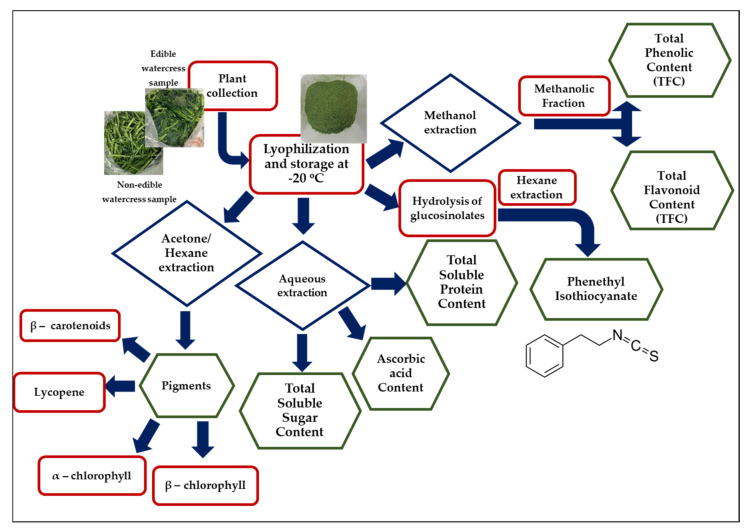
Graphic representation of optimized extraction procedures for the isolation of major nutrients and phytochemical compounds found in edible and non–edible parts of watercress.

Freeze-dried edible and non-edible watercress samples were macerated by utilizing different extraction protocols depending on the chemical nature of the examined phytochemical (Figure 1).

The first step of the experimental pipeline was to induce the hydrolysis of glucosinolates, and, most specifically, that of gluconasturtiin (it being the most abundant) in order to isolate and quantify PEITC, taking into consideration the major factors that can affect its hydrolysis, such as pH, temperature, duration and dilution. The extraction of PEITC was performed using conventional liquid–liquid extraction methodologies by employing a range of solvents with different polarities. The hydrophobic ITCs—hence, PEITC—were extracted from the aqueous hydrolytic mixture by an immiscible solvent of low polarity: hexane (Figure S1). The quantification of PEITC occurred via UPLC MS/MS with multiple reaction monitoring (MRM) transitions (Figure S2), using pure synthetic PEITC as a reference standard (Table S1 and Figure S3). Finally, the PEITC content (from each of the three fractions) was determined in order to identify the most potent extraction solvent (Table 1 and Table S2)

Quantification via UPLC MS/MS via multiple reaction monitoring (MRM) mode revealed a differential degree of effectiveness for each of the three solvents utilized (i.e., hexane, chloroform and ethyl acetate) in the extraction of PEITC. The yield of PEITC ranged from 895 ± 21.54 to 1695 ± 100.46 μg of PEITC/g of dry extract for edible watercress, whereas the yield ranged from 0.12 ± 0.05 to 1002 ± 94.21 μg of PEITC/g of dry extract for non-edible watercress samples. As can be observed, the highest PEITC extraction yield was obtained with hexane, thereby making it the most potent solvent out of all three solvents used, whereas the least amount of PEITC was extracted in chloroform. Finally, it was observed that the edible watercress samples contained a significantly higher concentration of PEITC when compared to the non-edible one.

### 2.2. Determination of Total Polyphenol (TPC) and Flavonoid (TFC) Contents in Edible and Non-Edible Watercress Extracts

**Figure 2:**
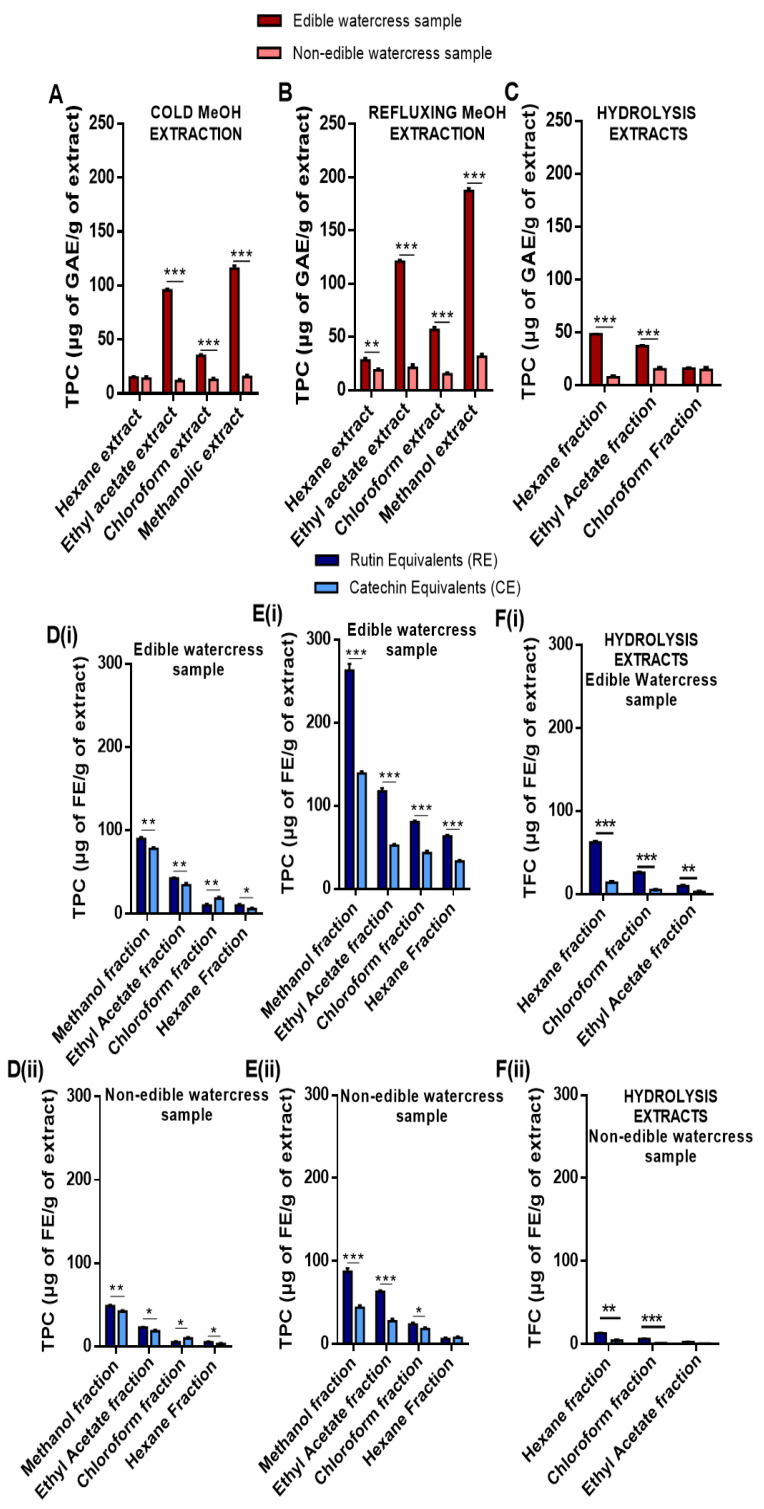
TPC and TFC profiles of both edible and non-edible watercress samples upon extraction with either refluxing or cold MeOH and subsequent fractionation with either MeOH or ethyl acetate or chloroform or hexane. TPC of each sample was quantified upon extraction with either (**A**) cold MeOH or (**B**) refluxing MeOH, whereas (**C**) represents the quantification of TPC from the hydrolysis of GLs in each sample. Data were expressed as gallic acid equivalents (GAE)/g of extract. On the other hand, TFC was quantified upon extraction of edible (**D**(**i**),**E**(**i**)) and non-edible (**D**(**ii**),(**E**(**ii**)) watercress samples with either refluxing or cold MeOH, respectively, whereas (**F**(**i**),**F**(**ii**)) represent the quantification of TFC from the hydrolysis of GLs in each sample, respectively. Data were expressed as rutin and catechin equivalents (RE and CE), respectively. All data are expressed as means ± SEM and are representative of three independent experiments. Statistical significance was set at * *p* < 0.05, ** *p* < 0.01, *** *p* < 0.001 upon one-way ANOVA analysis with Tukey’s test for multiple comparisons.

In another set of experiments, we sought to determine the total phenolic and flavonoid contents in both edible and non-edible watercress samples (Figure 2). Samples were macerated in the presence of aqueous methanol in either room or refluxing temperature, and then the extracts were fractionated in a range of solvents with different polarities. The results revealed that the same extraction and fractionation pattern is followed during the determination of the content of both phenolics and flavonoids (in both samples). Specifically, it appears that the extraction of both phenolics (TPC) and flavonoids (TFC) in either sample was more effective under refluxing temperature conditions, since a higher content of phenolics (Figure 2B) and flavonoids (Figure 2E(i,ii)) was extracted. On the contrary, the usage of MeOH at room temperature (Figure 2A,D(i,ii)) showed a reduced extraction capacity. Thus, it appears that methanol is the best solvent for the isolation of both phenolics and flavonoids compared to less polar solvents, such as ethyl acetate, chloroform and hexane (Figure 2A,B,E). Moreover, comparing the TPC and TFC obtained in both watercress samples, it appears that even the non-edible samples also contain phenolics and flavonoids, although at considerably lower concentrations (Figure 2A,B,D,E). Finally, both phenolic and flavonoid compounds were detected in the fractionated hydrolysis mixture of GLs in both samples, with the content of the non-edible sample being significantly lower (Figure 2F(i,ii)). In all cases, the highest concentration of TPC and TFC was noted in the hexane fraction.

### 2.3. Extraction of Total Soluble Proteins, Sugars and Ascorbic Acid, as Well as Various Pigments from Edible and Non-Edible Watercress Samples

**Figure 3:**
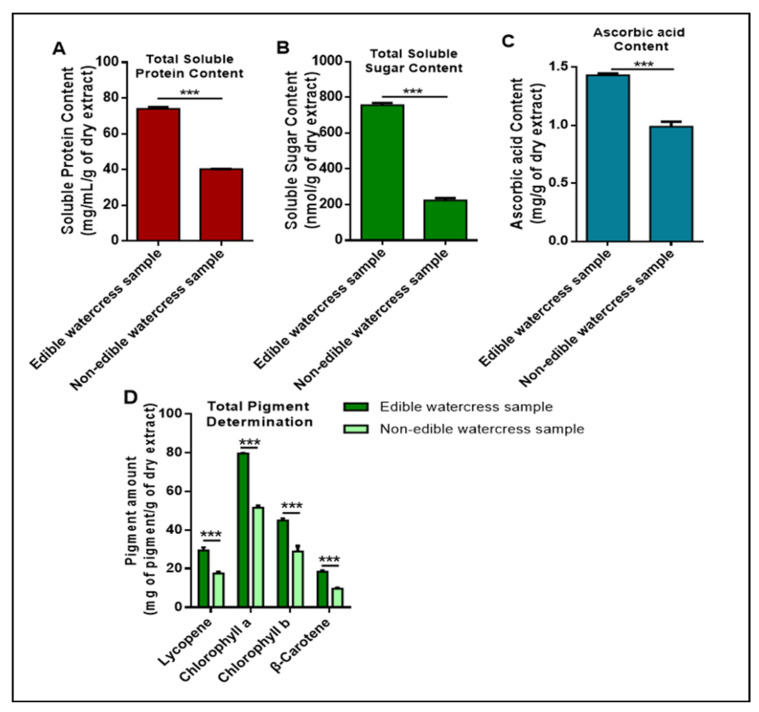
Profiling of various nutrients and phytochemicals in edible and non-edible watercress samples. Quantification of total soluble content of proteins (**A**) and sugars (**B**), together with total content of ascorbic acid (**C**) and pigments; (α-, β- chlorophylls, β-carotenoids and lycopene) (**D**). Data are expressed as means ± SEM and are representative of three independent experiments. Statistical significance is indicated by *** *p* < 0.001 upon one-way ANOVA analysis with Tukey’s test for multiple comparisons.

Next, by performing aqueous extraction, we were able to detect and quantify the total content of soluble proteins and sugars, as well as ascorbic acid in edible and non-edible watercress samples (Figure 3A–C), respectively.

In general, it was observed that the edible sample contains significantly higher levels of soluble proteins and sugars, as well as ascorbic acid. In addition, by acetone/hexane mixture extraction, we were able to quantify the major pigments with significant antioxidant activity (Figure 3D). Particularly, it can be demonstrated that α– chlorophyll levels are considerably higher compared to those of β– chlorophyll, whereas lycopene and β–carotenoids exist in much smaller concentrations. The profile of all pigments, in both watercress samples, follows the same trend regardless of the differences in relative magnitude; that is, the levels of pigments in the edible watercress sample are significantly higher than in the non-edible one.

The same analyses were also performed in the PEITC/phenolics/flavonoids enriched hexane fraction obtained from the hydrolysis of GLs in both watercress samples (Table 2).

As can be observed from Table 2, the hexane fraction of the edible watercress sample contains higher concentrations of all components (compared to the respective fractions of non-edible watercress samples), except for TSPC. In general, among total phenolics, rutin equivalents appear to make up the highest proportion in the edible sample, as opposed to the non-edible one, where catechin seems to be the predominant form. In addition, in the case of the total pigments content, β– chlorophyll appears to be the predominant pigment found in the hexane fraction of both watercress samples. Finally, in both samples, β–carotene was present as the least quantity among all pigments tested.

### 2.4. Antioxidant Evaluation of Edible and Non-Edible Watercress Extracts

**Figure 4:**
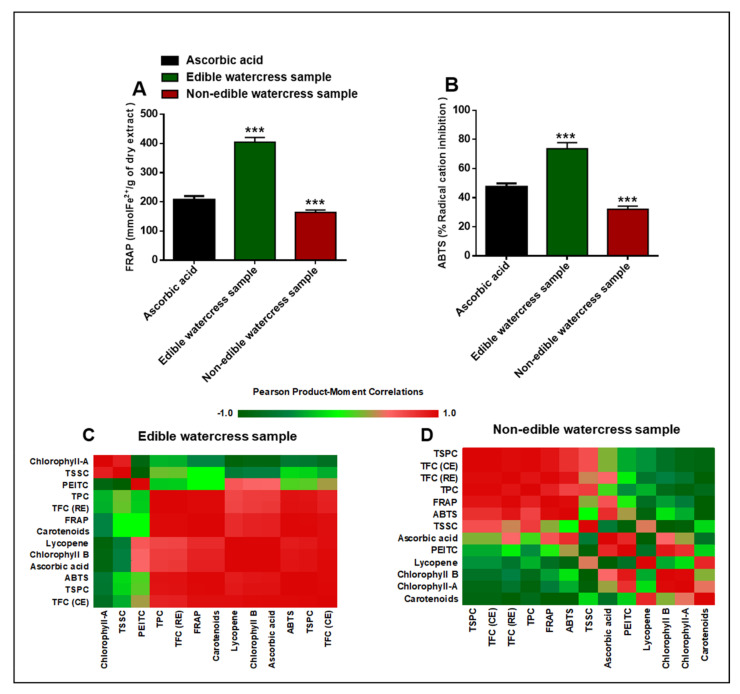
Cell-free antioxidant activity levels in both edible and non–edible watercress samples, as determined by the (**A**) FRAP and (**B**) ABTS assay kits. Ascorbic acid (at 1 mg/mL) was used as a reference standard. Data are expressed as means ± SEM and are representative of three independent experiments. Statistical significance is indicated by *** *p* < 0.001 upon one-way ANOVA analysis with Tukey’s test for multiple comparison. Additionally, Pearson’s correlation analysis was determined between major nutrients and phytochemical compounds with antioxidant activity levels in both edible (**C**) and non-edible (**D**) watercress samples. Red indicates a positive correlation (+1.0), whereas bright and dark green denote no correlation or negative correlation (−1.0), respectively.

The analysis of the antioxidant capacity of each hexane fraction in each of the watercress samples has led to significant differences in terms of the antioxidant potential of each sample, as determined by the ferric reducing antioxidant power (FRAP) (Figure 4A) and 2-2′-azino-bis-3-ethylbenzothiazoline-6-sulfonic acid (ABTS) assays (Figure 4B). Overall, it appears that the highest FRAP and ABTS values were measured in the hexane fractions obtained from the edible watercress samples, as opposed to the respective significantly lower values obtained from the non-edible ones (Figure 4A,B). Additionally, the degree of linearity (Pearson’s correlation) between some of these compounds and the levels of relevant antioxidant activity (as determined by the ABTS and FRAP assays) was illustrated by means of a heatmap. The Pearson’s correlation analysis indicated significantly positive correlations between PEITC, TPC, TFC (including CE and RE), TSPC, carotenoids, ascorbic acid, lycopene and β–chlorophyll (all extracted from the hexane fraction of edible samples) and FRAP/ABTS activity levels (Figure 4A,B). In contrast, the total soluble contents of sugars, proteins and α–chlorophyll showed a negative correlation with FRAP/ABTS activity levels. Finally, in the case of non–edible samples, a strong correlation was noted between TSPC, TFC (CE and RE), TPC, TSSC, ascorbic acid and FRAP/ABTS activity levels (Figure 4C). However, in this case, TSSC, PEITC, lycopene, α– and β– chlorophylls and carotenoid contents were negatively correlated with FRAP/ABTS activity levels.

### 2.5. Biological Evaluation of Edible and Non-Edible Watercress Extracts

**Figure 5:**
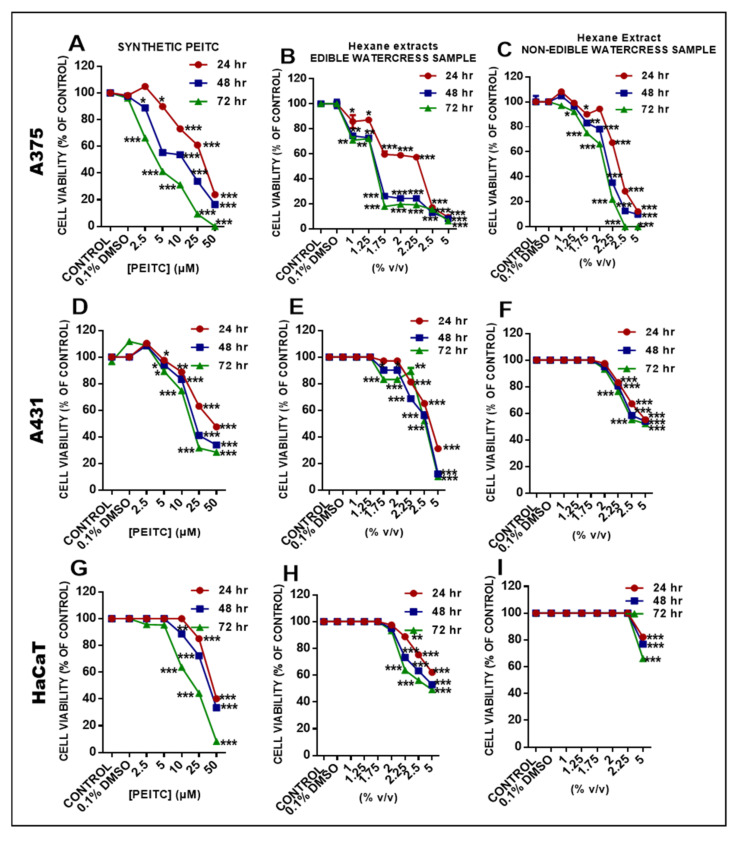
Cytotoxicity profiles of synthetic PEITC, as well as edible and non-edible watercress samples, in a human experimental model of malignant melanoma consisting of primary malignant melanoma cells (A375) (**A**–**C**), non-melanoma epidermoid carcinoma cells (A431) (**D**–**F**) and non-tumorigenic immortalized keratinocytes (HaCaT) (**G**–**I**) exposed to either 2.5–50 µΜ of synthetic PEITC or 1–5% (*v*/*v*) of edible and non-edible watercress extracts over 24, 48 and 72 h. Data are expressed as means ± SEM and are representative of three independent experiments. n.d represents data not determined. Statistical significance is indicated by * *p* < 0.05, ** *p* < 0.01, *** *p* < 0.001 relative to corresponding (0.1% DMSO) controls.

Moreover, we evaluated the anticancer potency of both watercress samples in an in vitro model of human malignant melanoma consisting of primary malignant melanoma (A375) (Figure 5A–C), non-melanoma epidermoid carcinoma (A431) (Figure 5D–F) and non-tumorigenic keratinocyte (HaCaT) (Figure 5G–I) cells. Overall, it was demonstrated that treatment with the edible watercress sample significantly decreased the viability levels of A375 cells in a concentration- and time-dependent manner. Specifically, the EC_50_ value at 48 h appears to be much lower than the respective EC_50_ value at 24 h, and at the same level as that at 72 h. This pattern was found to be of the same order but of a different magnitude when compared to those of non-edible watercress samples and synthetic PEITC (Table 3). For the evaluation of the cytotoxic potency of the lipophilic extract (obtained from both edible and non-edible watercress samples), our data were expressed as follows. First, results were expressed as % *v*/*v* in order to demonstrate the cytotoxic effect of the whole fraction, including its different components. Second, in order to compare the cytotoxic potency of the synthetic PEITC with the PEITC content of the hexane fraction, the viability results were presented as the μM concentration of PEITC in the hexane fraction. Thus, the EC_50_ of the PEITC in the hexane fraction was estimated based on the total PEITC found in that fraction in both edible and non-edible watercress samples.

A time- and concentration-dependent pattern of EC_50_ values was also observed in A431 cells, but at much higher EC_50_ values compared to those in A375 cells, indicating their resistance to the effects of edible and non-edible watercress, as well as synthetic PEITC samples. In contrast, HaCaT cells were very minimally affected and, as such, the effect of all samples tested did not appear to follow any specific pattern of cytotoxicity induction (Table 3).

**Figure 6:**
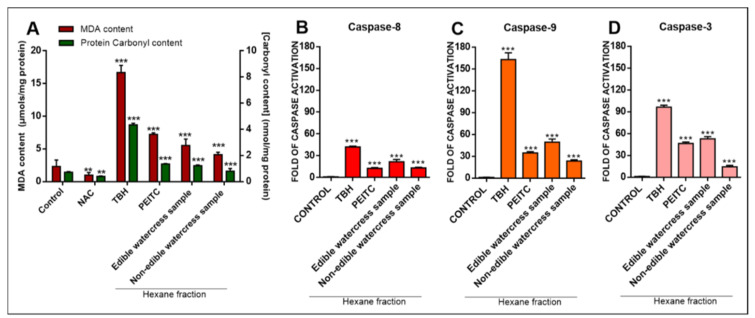
The effect of edible and non-edible watercress samples on lipid and protein oxidation levels in human malignant melanoma (A375) cells. (**A**) Lipid and protein oxidation levels (MDA and carbonyl contents, respectively) were determined upon treatment of A375 cells with either 2.5 mM *N*-acetyl cysteine (NAC; negative control),200 μΜ *tert*-butyl hydroperoxide (TBH; positive control), 10μM synthetic PEITC or 1.3% (*v*/*v*) edible or 2.5% (*v*/*v*) non-edible watercress samples, for 48 h of exposure. On the other hand, caspases−8 (**B**), −9 (**C**) and −3 (**D**) activities were determined in A375 cells following exposure to 1.3% (*v*/*v*) edible or 2.5% (*v*/*v*) non-edible watercress samples for 48 h. As positive control, 200 μM of TBH was used. Data shown are means ± SEM of and are representative of three independent experiments. Asterisks (**) denote statistical significance when compared to their respective control at *p* < 0.01 whereas *** denote statistical significance at *p* < 0.001.

Given that the most effective experimental conditions of exposure were shown to be at 48 h, A375 cells were subjected to either 10 μM synthetic PEITC or 1.3% (*v*/*v*) edible or 2.5% (*v*/*v*) non-edible watercress samples (their corresponding EC_50_ values). Following the determination of intracellular levels of lipid and protein oxidation (MDA and carbonyl contents, respectively) by commercially available kits, it was shown that exposure to TBH (200 μM) significantly increased intracellular levels of MDA and carbonyl contents to a higher degree compared to the rest of the tested samples. Specifically, although exposure to edible or non-edible watercress samples, as well as synthetic PEITC, also led to significant increases in both contents (when compared to the control), such increases were not of the same magnitude when compared to TBH (Figure 6A).

Finally, under the same experimental conditions of exposure, all tested samples were shown to be capable of inducing the apoptotic response measured as caspases −3, −8 and −9 activities by utilizing a commercially available multiplex assay kit. Specifically, our data revealed that, under each experimental condition, the levels of caspases −9 and −3 were induced at much higher levels (Figure 6C,D, respectively), as opposed to those of caspase −8 (Figure 6B), highlighting the importance/involvement of mitochondria in modulating the induction of the intrinsic apoptotic response.

## 3. Discussion

The present study aims to assess the overall health-promoting impact of *Nasturtium officinale* (watercress) plant extracts by characterizing some of the major nutrients and phytochemicals found in both edible (leaves and lateral bud) and non-edible (stems) parts of the plant biomass, namely, PEITC, total phenolics, total flavonoids, various pigments, total sugars, total proteins and ascorbic acid. Additionally, the current work evaluates the biological potency of watercress extracts by being able to trigger antioxidant, cytotoxic, oxidative stress and apoptotic induction responses against an in vitro model of human malignant melanoma.

Taking into consideration the factors that can affect the hydrolysis of GLs, and hence the effective production of PEITC, we followed a methodology similar to Rodrigues et al. (2016), according to which, the optimum temperature for myrosinase activity varied between 25–45 °C, whereas the pH was kept between 7 and 9 [32]. In addition, the utilization of hexane as a solvent for the conventional extraction of PEITC from watercress has been previously suggested by other studies [32,33,34]. Herein, we were able to extract similar quantities to those previously reported by others [33,34]. More specifically, Coscueta et al. (2020) claimed that the extraction of PEITC with hexane led to the isolation of 1682 ± 156 μg of PEITC/g of dry watercress, whereas Farhana et al. (2016) extracted 2300 μg of PEITC/g of dry watercress [33,34]. These differences in the amount of extracted PEITC are strongly correlated with several factors, including cultivation conditions (soil pH, temperature and watering frequency), plant species and age [14,35,36]. To the best of our knowledge, this is the first report that provides evidence for the existence of PEITC in watercress stems, considered to be watercress waste by-products, in addition to other well-known edible sources (i.e., leaves and lateral bud) of the plant. Many studies in the literature have identified and quantified phenolic and flavonoid compounds present in watercress leaves and stems [31,33,36]. Specifically, it has been previously suggested that watercress contains a high content of polyphenols, such as kaempferol-3 (feroyl-triglucoside) 7-rhamnosyl and quercetin 3- (para coumaroyltruglucoside) 7- rhamnosyl, in addition to flavonoids, such as quercetin 3-triglucoside-7 rhamnoside, kaempferol 3-triglucosie-7 rhamnoside and cinnamoyl acylated derivatives [33,37,38,39]. Our data demonstrated that the use of a hydro-alcoholic solvent system was considerably more efficient in the extraction of phenolics and flavonoids compared to other polar and non-polar solvents [38,39]. According to the literature, the potency of hydro-alcoholic solvents in effectively extracting phenolic and flavonoid compounds can be attributed to the dipole–dipole interactions developed between the hydroxyl groups of both phenolic and flavonoid compounds, thereby increasing their solubility [40,41,42]. Moreover, work by other groups has documented the significant differences in the nutritional content among different parts of various plants [43,44]. Similarly, watercress leaves and lateral buds contain increased levels of phytochemicals compared to stems, petioles and roots [43,44]. To this end, our findings (regarding the content of pigments) are consistent with the literature in that we have provided evidence that the edible parts of watercress (i.e., leaves and lateral buds) contained significantly higher amounts of chlorophyll-α and -β, lycopene and β-carotenoids compared to the non-edible parts of the plant (i.e., stems) [33]. The fact that the majority of the pigments have characteristic antioxidant activity ensures that the hexane fraction (which has been used throughout this study) is, in fact, supplemented with additional antioxidant components besides PEITC. In addition, both chlorophylls-α and -β were found to be the most abundant pigments that belong among the most well-known natural antioxidant compounds [45,46,47]. Similarly, increased levels of phenolics, flavonoids, ascorbic acid and soluble sugars and proteins were found at a significantly higher content in the PEITC-enriched (hexane) fraction obtained from the edible watercress part when compared to the non-edible one. Interestingly, the total content of soluble proteins was found to be much higher in the PEITC-enriched (hexane) fraction obtained from the non-edible watercress part, as opposed to that derived from the edible one (probably owing to its large content of stems). Overall, our data are in agreement with the results of other reports indicating that PEITC-enriched fractions (obtained by the extraction of the hydrolysis of GLs) contained, among others, phenolic compounds, flavanols, hydroxycinnamic acid derivatives and traces of ascorbic acid [32].

Finally, an evaluation of the antioxidant capacity was performed by means of employing both FRAP and ABTS assays. The findings from both assays were consistent with each other for both edible and non-edible watercress samples. Consequently, it appears that the content of both extracts was capable of acting as antioxidant compounds. More specifically, the hexane fraction obtained from the edible watercress sample was considerably more potent in inhibiting radical cation formation (73.65 ± 4.16% radical cation inhibition), as well as reducing Fe^3+^ into Fe^2+^ (404.25 ± 16.31 mmol of Fe^2+^/g of dry extract), when compared to ascorbic acid (reference standard; 47.54 ± 2.35% radical cation inhibition and 207.43 ± 12.25 mmol of Fe^2+^/g of dry extract) in both FRAP and ABTS assays, respectively. Similarly, the hexane fraction obtained from the non-edible watercress was also shown to possess antioxidant activity (163.78 ± 8.21 mmol of Fe^2+^/g of dry extract and 32.11 ± 2.11% radical cation inhibition) in both FRAP and ABTS assays, respectively; however, it was of a lower magnitude when compared to either ascorbic acid alone or the hexane fraction from the edible watercress sample. Through Pearson’s analysis, the majority of the analytes (phenolics, flavonoids, ascorbic acid, lycopene, β–carotenoids and β– chlorophyll) exhibit a strong correlation with both FRAP and ABTS assays, suggesting their potency as antioxidant agents. On the other hand, α–chlorophyll, PEITC and soluble sugar contents were negatively correlated with antioxidant activity measured by both assays. Moreover, whereas TPC showed the strongest correlation with the antioxidant activity among all of the other phytochemicals, this association was not evident for PEITC, probably because ABTS and FRAP are cell-free-based assays and thus there is no participation of any type of metabolic enzymes that could account for any potential antioxidant activity. In addition, other studies have suggested that PEITC can act as a pro-oxidant compound during its metabolism through the formation of conjugates with glutathione (GSH), causing its depletion [24,48,49]. Furthermore, there was no observed correlation between the soluble sugars’ content and antioxidant activity. Interestingly, it has been suggested that soluble sugars are closely related with plant resistance to stress conditions, as the levels of soluble sugars increase during plant growth, whereas they decrease during adverse events, such as plant injury [50,51]. In this context, the reduction in soluble sugar levels during stress events can be attributed to the fact that they are used as building blocks for the biosynthesis of antioxidant compounds (e.g., phenolics, flavonoids, etc.), a response initiated as an adaptive mechanism against stress conditions [52,53,54].

The biological evaluation of both edible and non-edible watercress samples revealed a significant reduction in viability levels in A375 cells, followed by an increased cytotoxic effect in A431 cells (however, requiring higher EC_50_ values) and ultimately a minimum effect on HaCaT cell viability levels. In addition, given that PEITC represents the major component of the total phytochemical content in both watercress samples (to a variable degree), exposure to synthetic PEITC was also utilized in an attempt to compare its bioactivity profile (i.e., cytotoxicity, generation of oxidative stress, apoptotic induction) to that of both naturally occurring watercress samples. Our results relevant to the cytotoxic effect of synthetic PEITC were consistent with previously published data [55]. As expected, the exposure of all cell lines to the edible watercress sample exhibited a higher cytotoxic potency, which is probably attributed to the substantial presence of significant contents of other phytochemicals, including flavonoids, phenolics, chlorophyll-b and ascorbic acid, all of which can act synergistically in potentiating the anticancer ability of such a sample. According to our results, a similar pattern of cytotoxicity was observed in all three cell lines tested, although to a different magnitude, as a progressive decline of EC_50_ values from 24 to 72 h of exposure was observed, with the most dramatic decline occurring at 48 h. Next, we sought to determine the effect of both types of watercress samples on the intracellular levels of lipid (MDA) and protein (carbonyl groups) oxidation in A375 malignant melanoma cells. According to the data analysis, all watercress samples were capable of increasing the levels of both lipid and protein oxidation, suggesting, at least in part, a potential pro-oxidant activity as an underlying, mechanism of the observed anticancer potency. Malignant melanoma cells are characterized by a dysregulated redox state [56,57,58,59] with significantly higher basal levels of reactive oxygen species (ROS) compared to those of keratinocytes and fibroblasts [59,60,61]. In addition, it is reported that high levels of ROS are essential for the progression and transformation of melanoma cells, as the inhibition of ROS accumulation leads to the suppression of their metastatic potential, as evidenced in an in vivo experimental model [62]. However, it is also reported that an excessive accumulation of ROS can also have a detrimental effect for melanoma cells, since several apoptotic cascades are activated, eventually leading to cell death [63,64]. In this context, we have determined the potential of watercress samples to induce apoptotic cell death as a response to previously induced levels of lipid and protein oxidation, further characterizing their anti-melanoma properties, through the activation of caspases −3 (execution caspase), −8 (marker of extrinsic apoptotic induction) and −9 (marker of intrinsic apoptotic induction) expression levels. Our data have shown that both watercress samples have the capacity to induce an apoptotic response, in a manner similar to that observed in oxidative stress generation, where the edible samples showed a greater degree of potency compared to that of the non-edible ones. Either way, it appeared that the apoptotic response was at least partially dependent on the activation of caspase −9, thereby indicating a potential role of the intrinsic apoptotic pathway. Finally, our data are in agreement with the work of other groups, highlighting the role of ITCs and particularly PEITC (as well as cruciferous vegetables as a whole) in inhibiting cancer growth through apoptotic induction [24,39,65,66,67,68,69,70].

## 4. Materials and Methods

### 4.1. List of Reagents

Solvents: hexane ≥ 97% (Honeywell, *Cat No*. 34859, Medisell, Cyprus), chloroform ≥ 99.8% (Honeywell, *Cat No*. 319988, Medisell, Nicosia, Cyprus), ethyl acetate ≥ 99.5% (Honeywell, *Cat No*. 33211, Medisell, Nicosia, Cyprus), methanol HPLC grade ≥ 99.9% (Honeywell, *Cat No*. 34860, Medisell, Nicosia, Cyprus), water HPLC grade (Honeywell, *Cat No*, 34877, Medisell, Nicosia, Cyprus), acetonitrile HPLC grade ≥ 99.9 (Honeywell, *Cat No*. 34851, Medisell, Nicosia, Cyprus), sulfuric acid 95–97% (Sigma Aldrich, *Cat No*. 10009731, Saint Louis, MO, USA), acetone ≥ 99.8% (Honeywell, *Cat No*, 34580, Medisell, Nicosia, Cyprus), trifluoroacetic acid (TFA) LC-MS grade (Thermofisher, *Cat No*. 85183, Medisell, Nicosia, Cyprus), acetic acid ≥ 99.7% (Sigma Aldrich, *Cat No*. 6950992, Saint Louis, MO, USA). Analytical standards and reagents: PEITC standard (Sigma Aldrich, *Cat No*. 68488, Saint Louis, MO, USA), ascorbic acid standard (Sigma Aldrich, *Cat No*. PHR1008, Saint Louis, MO, USA), TBA malondialdehyde (MDA) standard (Cayman, *Cat No*.10009202, Ann Arbor, MI, USA), 2,4-dinitro phenyl hydrazine (2,4-DNPH) (Cayman, *Cat No*. 10009202, Ann Arbor, MI, USA), gallic acid standard (Sigma Aldrich, *Cat No.* 91215), rutin standard (Sigma Aldrich, *Cat No.* 78095, Saint Louis, MO, USA), catechin standard (Sigma Aldrich, Cat No. 43412, Saint Louis, MO, USA), mannose (Sigma Aldrich, *Cat No.* 92683, Saint Louis, MO, USA), *N*-acetyl cysteine (NAC) (Fluorochem, *Cat No*. 003631, Derbyshire, UK), resazurin sodium salt (Fluorochem, *Cat No*. 492502, Derbyshire, UK), guanidine hydrochloride (Fluorochem, *Cat No*. 044943, Derbyshire, UK), dimethyl sulfoxide (DMSO) (PanBiotech, *Cat No*. P60-36720100, Lab Supplies, Athens, Greece). Cell culture reagents: fetal bovine serum (FBS) (heat inactivated) (BIOSERA, *Cat No. FB-1001H/500*, Lab Supplies, Athens, Greece) penicillin/streptomycin (pen/strep) (BIOSERA, *Cat No*. LM-A4188/100, Lab Supplies, Athens, Greece), DMEM high glucose media w/o L-glutamine w/sodium pyruvate (BIOSERA, *Cat No*. LM-D1112/500, Lab Supplies, Athens, Greece), Dulbecco’s phosphate buffer saline (PBS) *w*/*o* calcium, *w*/*o* magnesium (BIOSERA, *Cat No*. LM-S2041/500, Lab Supplies, Athens, Greece), L-glutamine (BIOSERA, *Cat No*. XC-T1715/500, Lab Supplies, Athens, Greece), trypsin-EDTA 100× (BIOSERA, *Cat No*. XC-TC1717/100, Lab Supplies, Athens, Greece).

### 4.2. Plant Material Cultivation, Processing and Storage

Fresh edible and non-edible watercress samples were kindly provided by The Watercress Company, Dorchester, Dorset, UK. The aerial parts of watercress plants were kept at −20 °C until further use. Watercress parts were sprayed with liquid nitrogen prior to drying them in a freeze-drier (Christ Alpha 1-4, LSC Basics) at −55 °C, 0.05 m bar for 96 h. The de-hydrated parts were then re-immersed in liquid nitrogen and milled to a fine powder using a domestic blender. The freeze-dried watercress powdered samples were kept at −20 °C in a sealed bag protected from air, humidity and light until further use.

### 4.3. Hydrolysis of Glucosinolates, Extraction and Quantification of PEITC

The hydrolysis of glucosinolates was performed according to Rodrigues et al. (2016) with some modifications [32]. Briefly, the hydrolysis was promoted by stirring five (5.0) g of each of the freeze-dried watercress samples with 315 mL of phosphate buffer (PBS) (pH 7.4) for 2 h at 37 °C. The hydrolysis solution mixture was extracted at 37 °C for 2 h with a range of solvents, including hexanes, chloroform and ethyl acetate. The resulting bilayer mixture was filtered through a Whatman paper (pore size: 4.0–12.0 µm) and the two layers were isolated using a separating funnel. The organic layer was dried over magnesium sulfate and concentrated to dryness at 40 °C under reduced pressure. The isolated extracts were kept at −20 °C until further use. For the quantification of PEITC, a Waters Acquity UPLC system equipped with a triple-quadrupole tandem mass spectrometer (Xevo TQD), with an autosampler chamber, was used for UPLC MS/MS analysis. The chromatographic separation was performed on a BEH C18 (2.1 × 50 mm, particle size: 1.7 μm) column (Waters, Acquity UPLC—Milford, MA, USA) heated at 35 °C. The mobile phase consisted of a solution of TFA 0.1% (*v*/*v*) (eluent A) and methanol 100% (eluent B). A flow rate of 0.1 mL.min^−1^ was used, and the gradient conditions applied consisted of 3% B (0–5 min), 3–40% B (5–10 min), 40–60% B (10–15 min), 60–70% B (15–20 min), 70–80% B (20–21 min), 80% B (21–22 min), 80–40% B (22–25 min), 40–3% B (25–26 min). The injection volume was 10 μL. MS/MS experiments were performed using a Xevo TQD mass spectrometer with an electrospray in positive ion mode (ESI+). Analytical conditions were optimized to maximize the precursor ion signal [M+H]^+^ with the ion source at 149 °C. The analyte was ionized and spectra of the column eluate were monitored in the ‘Full Scan’ mode in a range of *m*/*z* 100–1000. For the MS/MS experiments, the applied collision energy in the in-source collision-induced dissociation mode was 22 eV and the collision energy in the MS/MS mode was set at 30 eV. High-purity nitrogen gas was used as the drying and nebulizing gas, whereas ultrahigh-purity argon was used as a collision gas. The quantification was accomplished using selected reaction monitoring for the transitions: PEITC, [M+H]^+^, *m*/*z* 164→*m*/*z* 130.0. MassLynx software (version 4.1) was used for data acquisition and processing. Finally, a stock solution of PEITC standard (≥99% purity) at a concentration of 1000 ppm in acetonitrile was prepared and diluted (with acetonitrile) to a series of working standard solutions. The calibration curve ranged from 2ppb to 100 ppm (R^2^ > 0.998). QC samples were prepared at five (5.0) different concentrations of the lower level of quantitation quality control (LLOQC, 2 ppb), low-quality control (LQC, 500 ppb), medium-quality control (MQC, 50 ppm) and high-quality control (HQC, 100 ppm). Stock and working solutions were kept at 4 °C.

### 4.4. Determination of Total Flavonoid and Phenolic Contents

For the determination of total flavonoid (TFC) and phenolic (TPC) content, five (5.0) g of each of the freeze-dried watercress samples were extracted by maceration with aqueous methanol (70% *v*/*v*) (either in refluxing or room temperature) for 48 h. The resulting extraction mixtures were diluted by the addition of double distilled (dd) water at a volume of 10% of the total volume of methanol. The solutions were filtered through a Whatman filter paper (pore size: 4.0–12 μm) and fractionated with a range of solvents, including hexane, chloroform and ethyl acetate. The organic layers (separately) were isolated, dried and concentrated under reduced pressure. For the determination of TFC, the resulting fractions were taken up in acetonitrile and kept at −20 °C until further use. For the quantification of TFC, 20 μL of each fraction was diluted with 60 μL of methanol and mixed with 10 μL of aluminum trichloride (10% aqueous solution) and 10 μL of sodium acetate (0.5 M aqueous solution). The resulting solutions were allowed to stand in the dark at room temperature (RT) for 40 min, and the absorbance was monitored at 415 nm. The TFC was determined based on both catechin (linear range: 0–100 μg/mL, R^2^ > 0.989) and rutin (linear range: 0–500 μg/mL, R^2^ > 0.995) calibration curve. The results were expressed as μg of catechin or μg of rutin equivalents/g of dry extract. For the determination of TPC, the resulting fractions were taken up in acetonitrile and the quantification was performed using a commercial polyphenolic quantification assay kit (Bioquochem, Asturias, Spain) according to the manufacturer’s instructions. The results were expressed as μg of gallic acid equivalents/g of dry extract.

### 4.5. Determination of Pigments

For the determination of pigments, five (5.0) g of each of the freeze-dried watercress samples were macerated with 100 mL of acetone/hexane mixture (4:6 *v*/*v*), which was stirred vigorously at RT for 10 h. Then, they were filtered through a Whatman filter paper (pore size: 4.0–12.0 µm), and their total contents in chlorophyll-a and -b, lycopene and β-carotene were determined as previously described [71]. The absorbance was measured sequentially at 453, 505, 645 and 663 nm using a microplate reader (LT4500, Labtech, UK), and the content was calculated using the following Equations (1)–(4):

Chlorophyll a (mg/g of dry extract) = [0.999 × A_663_ − 0.0989 × A_645_]/20 (1)

Chlorophyll b (mg/g of dry extract) = [1.77 × A_645_ − 0.328 × A_663_]/20 (2)

Lycopene (mg/g of dry extract) = [−0.0458 × A_663_ + 0.204 × A_645_ + 0.372 × A_505_ − 0.0806 × A_453_]/20 (3)

β-Carotene (mg/g of dry extract) = [0.216 × A_663_ − 1.22 xA_645_ − 0.304 × A_505_ + 0.452 × A_453_]/20 (4)

### 4.6. Determination of Total Soluble Protein Content

The total soluble protein content was determined, with some modifications, as previously described [72]. Briefly, fifty (50) mg of each of the freeze-dried watercress samples were soaked in 10 mL of dd water. The resulting suspension was stirred by vortexing at RT for 30 s. The suspensions were allowed to stand at RT for 30 min, and then they were centrifuged at 4000× *g* for 10 min. The protein content was determined by utilizing the BCA protein assay kit (Thermo Scientific, Waltham, MA, USA), according to the manufacturer’s instructions, and the absorbance was monitored at 562 nm using a microplate reader (LT4500, Labtech, Heathfield, UK). The soluble protein content was calculated based on a standard curve of bovine serum albumin (BSA) (linear range: 0–2 mg/mL, R^2^ > 0.997). The results were expressed as mg of protein/ mL/g of dry extract.

### 4.7. Determination of Total Soluble Sugar Content

Soluble sugar content was determined, with some modifications, as previously described [73]. Fifty (50) mg of each freeze-dried watercress samples were mixed with 10 mL of dd water and heated at 95 °C for 20 min. Homogenates were centrifuged at 4000× *g* for 10 min. Then, 150 μL of extracts were mixed with a concentrated solution of sulfuric acid, and the resulting solution mixture was shaken for 30 min at RT, followed by the addition of 30 μL of 5% phenol. The final mixture was heated at 90 °C for 5 min, and then it was allowed to cool at RT, before monitoring the absorbance at 490 nm. Total soluble sugar content was determined based on mannose calibration curve (linear range: 0–100 nM, R^2^ > 0.999). The results were expressed as nmol of mannose equivalents/g of dry extract.

### 4.8. Extraction and Quantification of Ascorbic Acid

Ascorbic acid content was determined, with some modifications, as previously described [74]. Fifty (50) mg of each freeze-dried watercress sample was extracted with 5 mL of 1% (*w*/*v*) of oxalic acid by continuous vortexing for approximately 20 min in order to minimize the autooxidation of ascorbic acid into dehydroascorbic acid. Homogenates were then centrifuged at 4000× *g* for 5 min. The supernatant was passed through a 0.45 μm cellulose acetate filter. Quantification of ascorbic acid content was performed on a Waters HPLC system with a Model 2998 photodiode array (PDA) detector (Waters Inc, Milford, CT, USA). Each sample was injected at a volume of 20 μL and separated chromatographically on a Waters Spherisorb C18 column (150 × 4.6 mm, particle size; 5 μm) at RT using an isocratic elution of 0.1% oxalic acid at a flow rate of 1 mL.min^−1^. The amount of ascorbic acid was estimated based on the absorbance value at 243 nm of standards of ascorbic acid (linear range: 0–200 mg of ascorbic acid R^2^ > 0.999). The results were expressed as mg of ascorbic acid/g of dry extract.

### 4.9. Determination of Antioxidant Activity

Determination of antioxidant activity by the ferric reducing antioxidant power (FRAP) assay, as well as the 2,2-azinobis (3-ethyl-benzothiazoline-6-sulfonic acid) (ABTS) assay, involved the resulting hexane fraction of both edible and non-edible watercress samples in a concentration of 1% *v*/*v* being diluted (1:100) and then quantified by using either a commercial FRAP or an ABTS Assay Kit (Bioquochem, Asturias, Spain) according to the manufacturer’s instructions. As a positive control, in both assays, ascorbic acid was used at a concentration of 1 mg/mL. For the FRAP assay, results were expressed as mmol of Fe^2+^/g of dry extract based on a calibration curve of Fe^2+^ standard (0–700 μM, linear range, R^2^ > 0.999). For the ABTS assay, results were expressed as percent radical cation inhibition based on a calibration curve of ascorbic acid standard (0–700 μM, linear range, R^2^ > 0.998). The percentage of radical cation inhibition was calculated according to Equation (5).

% Radical cation Inhibition = [1 − (Abs_f_/Abs_0_)] × 100 (5)

where Abs_f_ is the absorbance recorded at 734 nm, 5 min after the addition of samples, and Abs_0_ is the absorbance of the non-inhibited radical cation at 734 nm.

### 4.10. Cell Lines

The human malignant melanoma (A375) cell line was purchased from the American Type Culture Collection (ATCC, Manassas, VA, USA), whereas the human immortalized keratinocyte (HaCaT) cell line was kindly provided by Dr Sharon Broby (Dermal Toxicology & Effects Group; Centre for Radiation, Chemical and Environmental Hazards; Public Health England, Didcot, UK). Finally, the epidermoid carcinoma (A431) cell line was purchased from Deutsche Sammlung von Microorganismen und Zellkulturen (DSMZ—Braunschweig, Germany). A375 and HaCaT cells were cultured in DMEM high glucose media, whereas A431 cells were grown in RPMI media. Both types of culture media were supplemented with 10% fetal bovine serum (FBS), 2 mM L-glutamine and 1% pen/strep (100 U/mL penicillin, 100 μg/mL streptomycin). Cells were cultured in a humidified incubator at 37 °C and 5% CO_2_, grown as monolayers and sub-cultured at 80–90% confluency. All cell lines were cultured for 15–20 passages before new stocks were utilized. Finally, all media and reagents were purchased from Biosera (Kansas City, MO, USA), whereas all cell culture plastic was obtained from Corning (Corning, NY, USA).

### 4.11. Determination of Cell Viability

The Alamar Blue assay was utilized in this set of experiments. Briefly, cells were seeded in 100 μL/well into 96-well plates and incubated overnight prior to exposure to each of the watercress samples. The density of A375 cells was 8000, 4000, 2000 cells/well, and, for A431 and HaCaT cells, 10,000, 5000, 2500 cells/well for 24, 48 and 72 h, respectively. On the following day, cells were exposed to a range of concentrations [e.g., 1–10% *v*/*v* of each of the watercress samples (in 0.1% DMSO) and 2.5–50 μM of synthetic PEITC (also in 0.1% DMSO)] over 24–72 h of exposure. For control conditions, cells were incubated with complete medium only or 0.1% DMSO. At the indicated time points, 10 μL of resazurin [dissolved in PBS (1 mg/mL final concentration)] was added in each well and incubated for 4 h at 37 °C. The plates were then centrifuged, and the absorbance was recorded at 570 nm and 590 nm (reference wavelength) using a microplate reader (LT4500, Labtech, UK). The levels of cell viability were calculated and expressed as the percentage of control cells.

### 4.12. Determination of Malondialdehyde and Protein Carbonyl Contents

A375 cells were plated in 100 mm dishes (1.4 × 10^6^, 0.7 × 10^6^ and 0.4 × 10^6^ per dish for 24, 48 and 72 h, respectively) and cultured overnight. The next day, cells were treated with either *N*-acetyl cysteine (NAC) (2.5 mM), *tert*-butyl hydroperoxide (TBH) (200 μΜ), synthetic PEITC (28, 12 and 7 μM) or edible (16, 10 and 9 μM) or non-edible (32, 18 and 13 μM) watercress samples for 24, 48 and 72 h, respectively. After trypsinization, pellets were collected, re-suspended in PBS and sonicated. For the determination of lipid peroxidation content, the whole suspension was further diluted with 4 mL of 4% *v*/*v* acetic acid solution containing 8% TBA and 0.1% SDS. The final mixture was heated at 95 °C for 1 h and centrifuged at 3000 rpm for 2 min. The TBARS Assay kit (Cambridge Bioscience Ltd., Cambridge, UK) was utilized for the determination of malondialdehyde (MDA) content according to the manufacture’s protocol. For the determination of protein carbonyl content, cells were trypsinized and pellets were collected, re-suspended in PBS (supplemented with 1 mM EDTA) and sonicated. The Protein Carbonyl Colorimetric Assay Kit (Cambridge Bioscience Ltd., UK) was used according to the manufacture’s protocol.

### 4.13. Determination of Caspase Activity

Caspase activity was measured using the caspases −3, −8 and −9 multiplex assay kit (Abcam, UK). A375 cells were grown at a density of 4000 cells/well and were seeded in black 96-well plates. The following day, cells were treated with either TBH (200 μM) or edible (2 μΜ) or non-edible watercress sample (10 μM) for 48 h. At the end of the incubation period, a caspase loading solution was prepared according to the manufacturer’s protocol. Briefly, 50 μL of caspase substrate was mixed with 10 mL of assay buffer and, afterwards, 100 μL of caspase loading solution was added (without the removal of the culture medium) into each well. Cells were incubated for 1 h at RT (in the dark), and fluorescence intensity was measured using a fluorescence microplate reader (synergy H1, Bio-Tek, US) at Ex/Em = 535/620 nm for caspase 3, Ex/Em = 490/525 nm for caspase 8 and Ex/Em = 370/450 nm for caspase 9. Results were expressed as fold difference based on the average relative fluorescence intensity of the untreated control.

### 4.14. Statistical Analysis

Data were expressed as mean values ± standard deviation (SD) and comparisons were made between control and treated groups. Statistical analyses were performed by one-way ANOVA with Tukey’s test for multiple comparisons using the GraphPad Prism 6 software. Statistical significance was set at *p* < 0.05, *p* < 0.01 and *p* < 0.001. Pearson’s correlation analysis was made by heatmap generation using STATGRAPHICS 19 centurion (Statgraphics Technologies, Inc., The Plains, VA, USA).

## 5. Conclusions

Our results demonstrate, for the first time, that both edible and non-edible parts of watercress can have significant nutritional value (e.g., rich in isothiocyanates, polyphenols, phenolics, proteins, sugars, ascorbic acid, β-carotene, lycopene, etc.), in addition to exerting health-promoting and/or disease-preventing properties (e.g., being antioxidant). Particular interest should be paid to the non-edible watercress content, as we have shown that what is an otherwise waste by-product is actually a rich source of anticancer properties, as evidenced in our in vitro model of human malignant melanoma, and is therefore a biomass source of potential pharmaceutical interest.

## Figures and Tables

**Table 1:** PEITC content in edible and non-edible watercress samples extracted by various water immiscible solvents. ^a,b,c^ represent values of significant difference according to ANOVA analysis, with Tukey’s test at 0.05 level of significance.

Watercress Sample	Solvent	[PEITC] (μg/g of Dry Extract)
Edible	Hexane	1695 ± 100.46 ^a^
	Chloroform	895 ± 21.54 ^b^
	Ethyl Acetate	1023 ± 98.61 ^c^
Non-edible	Hexane	1002 ± 94.21 ^a^
	Chloroform	0.12 ± 0.05 ^b^
	Ethyl Acetate	1.23 ± 1.01 ^c^

**Table 2:** Composition of main nutrients and phytochemicals including PEITC, ascorbic acid, total soluble sugar content (TSSC), total soluble protein content (TSPC), pigments (α–, β– chlorophylls, lycopene and β–carotene), total phenolic content (TPC) and total flavonoid content (TFC). All of these compounds were contained in the hexane fraction of the hydrolysis of GLs mixture in both watercress samples, at various concentrations.

Hydrolysis of GLs—Hexane Fraction			Expression Units
Content	Edible Watercress Sample	Non-Edible Watercress Sample	
PEITC	1695 ± 100.46	1002 ± 94.21	μg/g dry watercress
Ascorbic acid	0.6021 ± 0.03	0.1015 ± 0.09	mg of ascorbic acid/g of dry extract
TSSC	212.45 ± 7.71	106.26 ± 2.36	nmol of mannose equivalents/g of dry extract
TSPC	15.98 ± 0.03	31.71 ± 0.09	mg of BSA equivalents/mL/g of dry extract
Pigments	14.22 ± 0.99	11.21 ± 2.66	mg of lycopene/g of dry extract
	16.82 ± 0.56	17.18 ± 1.69	mg chlorophyll–a/g of dry extract
	36.02 ± 2.13	25.93 ± 2.73	mg chlorophyll–b/g of dry extract
	0.077 ± 0.01	0.053 ± 0.002	mg β–carotene/g of dry extract
TPC	47.66 ± 0.63	9.31 ± 1.51	mg of gallic acid equivalent/g of dry extract
TFC	64.52 ± 2.69	12.94 ± 0.91	mg of rutin equivalents/g or dry extract
	13.55 ± 2.28	19.29 ± 1.88	mg of catechin equivalents/g of dry extract

**Table 3:** EC_50_ values [expressed either as % *v*/*v* or μM concentration of PEITC (where relevant)]) were estimated for all cell lines at each time point of exposure by utilizing the online EC_50_ calculator platform (Very Simple IC_50_ Tool Kit, available online: http://www.ic50.tk/ (accessed on 11 August 2021)).

		EC_50_ (% *v*/*v*) (μΜ PEITC)		
Cell Line	Time (h)	Synthetic PEITC (μΜ)	Edible Watercress Sample	Non-Edible Watercress Sample
A375	24	28.44 ± 1.12	2.31 ± 0.14	2.61 ± 0.11
			(9.99 ± 0.71 μM)	(24.13 ± 1.99 μM)
	48	11.66 ± 2.26	1.31 ± 0.10	2.54 ± 0.12
			(2.48 ± 0.11 μM)	(13.48 ± 1.19 μM)
	72	7.23 ± 1.38	1.21 ± 0.12	2.03 ± 0.15
			(1.78 ± 0.52 μM)	(6.27 ± 1.07 μM)
A431	24	23.22 ± 1.11	3.45 ± 0.21	4.89 ± 1.12
			(37.29 ± 2.42μM)	(42.10 ± 2.23 μM)
	48	19.48 ± 2.21	2.61 ± 0.13	4.82 ± 1.23
			(15.28 ± 1.06 μM)	(37.12 ± 1.77 μM)
	72	16.69 ± 1.37	2.64 ± 0.09	4.8 ± 1.11
			(17.42 ± 1.13 μM)	(36.01 ± 2.1 μM)
HaCaT	24	44.27 ± 1.2	n.d.	n.d.
	48	33.97 ± 1.27	4.96 ± 0.23	n.d.
			(45.42 ± 2.23 μM)	
	72	25.6 ± 2.11	4.92 ± 0.14	n.d.
			(38.92 ± 1.25 μM)	

## Data Availability

Data is available within the article and Supplementary Materials.

## Supplementary Materials

The following supporting information can be downloaded at: https://www.mdpi.com/article/10.3390/ph15020141/s1, Figure S1: Shows the ‘Full Scan’ chromatogram of the hydrolysis of glucosinolates solution extract of edible-watercress sample, prior the extraction with various solvents. The spectrum was recorded via Waters Acquity UPLC system equipped with a triple-quadrupole tandem mass spectrometer (Xevo TQD). PEITC can be detected at *m*/*z* = 164.21 in the positive ionization mode; Figure S2: The quantification of PEITC occurred via Multiple Reaction Monitoring (MRM) transitions; *m*/*z* 164 → 130. The parameters; cone voltage, collision energy, *m*/*z* of product ion as well as dwell were obtained from the Intellistar application of MassLynx Software when the precursor ion was set at 164.0 Da in the positive ionization mode; Figure S3: Calibration curved of PEITC standards (2 ppb-100 ppm) used for the quantification of the PEITC obtained from either edible and non-edible watercress sample; Table S1: Shows the area peak intensity obtained from different concentrations (2 ppb-100 ppm) of standard (PEITC) in acetonitrile that were used for the generation of calibration curve. Each sample was run 3 times and the average value of the area peak intensity was calculated; Table S2: Shows the area peak intensity of the different fraction of that were obtained from the hydrolysis of glucosinolates mixture of both edible and non-edible watercress mixture. The quantification of PEITC content was performed on three fractions obtained from three independed experiments calculated.
