# Supplementary material for: Evaluation of Bioactive Properties of Lipophilic Fractions of Edible and Non-Edible Parts of Nasturtium officinale (Watercress) in a Model of Human Malignant Melanoma Cells
Source: Pharmaceuticals (Basel). 2022 Jan 25;15(2):141. doi: 10.3390/ph15020141 (PMC8879096; doi:10.3390/ph15020141)
Supplement: Supplementary file 1 [file pharmaceuticals-15-00141-s001.zip › pharmaceuticals-1472398-supplementary.pdf]

Evaluation of Bioactive Properties of Lipophilic Fractions of Edible and non-Edible Parts of *Nasturtium officinale* (watercress) in a Model of Human Malignant Melanoma Cells

Sotiris Kyriakou[1,2], Venetia Tragkola[1,2], Heba Alghol[2], Ioannis Anestopoulos[1,2], Tom Amery[3], Kyle Stewart[4], Paul G. Winyard[4], Dimitrios T. Trafalis[5], Rodrigo Franco[6,7], Aglaia Pappa[8], Mihalis I. Panayiotidis[1,2*]

[1] Department of Cancer Genetics, Therapeutics & Ultrastructural Pathology, The Cyprus Institute of Neurology & Genetics, Nicosia, 2371, Cyprus; sotirisk@cing.ac.cy, venetiat@cing.ac.cy, ioannisa@cing.ac.cy, mihalisp@cing.ac.cy
[2] The Cyprus School of Molecular Medicine, Nicosia, 1683, Cyprus; sotirisk@cing.ac.cy, algholh@cing.ac.cy, venetiat@cing.ac.cy, ioannisa@cing.ac.cy, mihalisp@cing.ac.cy
[3] TheWatercress Company, Dorchester, DT2 8QY, UK; tom.amery@thewatercresscompany.com;
[4] Watercress Research Limited, Devon, TQ12 4AA, UK; kyle@watercress-research.com; paul@watercress-research.com
[5] Laboratory of Pharmacology, Medical School, National & Kapodistrian University of Athens, Athens, 11527, Greece; dtrafal@med.uoa.gr
[6] Redox Biology Centre, University of Nebraska-Lincoln, Lincoln, NE 68583, USA; rodrigo.franco@unl.edu
[7] Department of Veterinary Medicine & Biomedical Sciences, University of Nebraska-Lincoln, Lincoln, NE 68583, USA; rodrigo.franco@unl.edu
[8] Department of Molecular Biology & Genetics, Democritus University of Thrace, Alexandroupolis, 68100, Greece; apappa@mbg.duth.gr
* Correspondence: mihalisp@cing.ac.cy; Tel.: +357-22392626

Supplementary Material

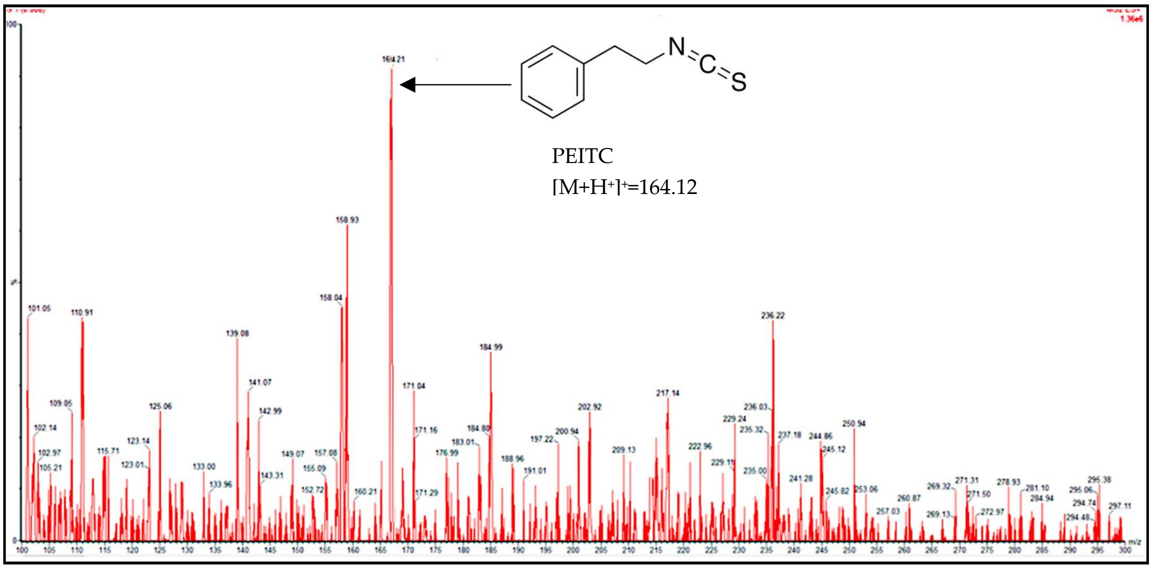

Figure S1: A ‘Full Scan’ chromatogram representative of the hydrolysis of glucosinolates’ solution extract, from the edible-watercress sample, prior to extraction with various solvents. The spectrum was recorded via Waters Acquity UPLC system equipped with a triple-quadrupole tandem mass spectrometer (Xevo TQD). PEITC can be detected at m/z=164.21, in the positive ionization mode.

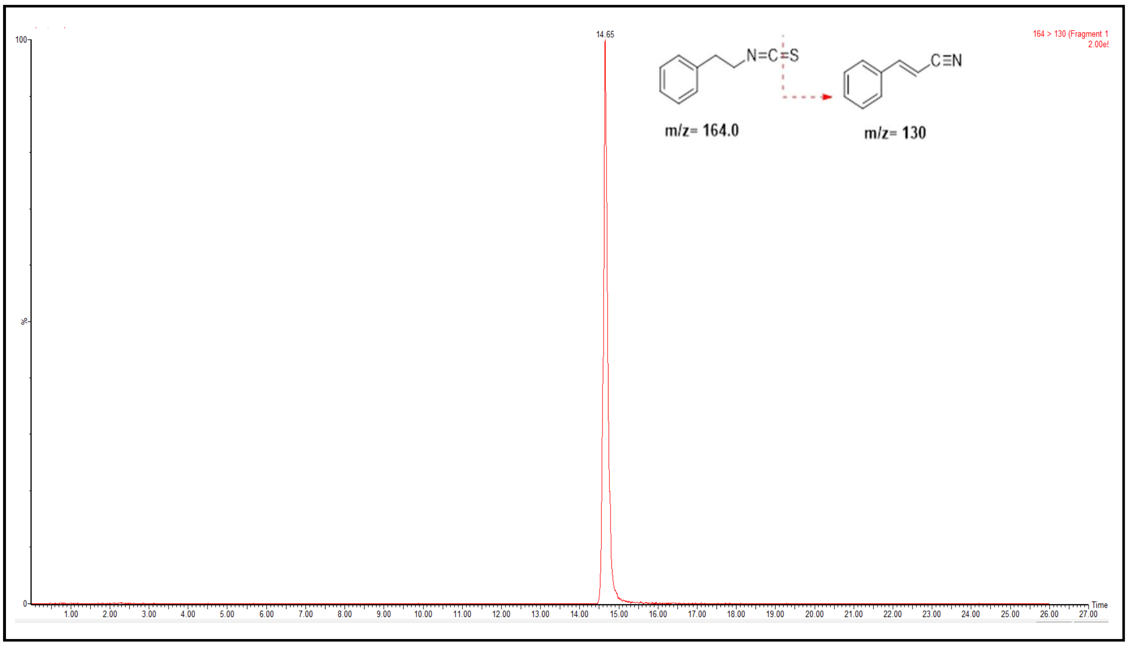

Figure S2: Quantification of PEITC content via Multiple Reaction Monitoring (MRM) transitions; m/z 164→130. The parameters; cone voltage, collision energy, m/z of product ion as well as dwell were obtained from the Intellistar application of

MassLynx Software when the precursor ion was set at 164.0 Da, in the positive ionization mode.

Table S1: Area peak intensity of the calibration curve obtained from different concentrations (2 ppb-100 ppm) of standard PEITC in acetonitrile solution. Each sample was run 3 times and the average value of the area peak intensity was calculated.

[PEITC] (ppb)	Run-1	Run-2	Run-3	Average	SD
2	424.967	468.239	451.235	448.147	21.801
4	760.200	753.215	787.165	766.860	17.928
5	918.320	925.235	920.215	921.257	3.573
10	1750.920	1730.258	1728.256	1736.478	12.547
20	3362.120	3356.847	3340.156	3353.041	11.466
50	8201.720	8163.215	8158.326	8174.420	23.768
100	16524.720	16830.000	16223.210	16525.977	303.397
200	32436.720	32340.330	32756.550	32511.200	217.877
500	81672.720	80668.160	80979.340	81106.740	514.255
1000	161232.700	161228.200	161235.511	161232.137	3.688
2000	362352.700	402342.100	302312.214	382347.400	28276.776
5000	815712.800	805709.300	790721.300	804047.800	12578.323
10000	1611313.000	1611309.000	1611324.141	1611315.380	7.846
100000	16435110.012	13122110.000	13110000.321	14222406.778	916266.778

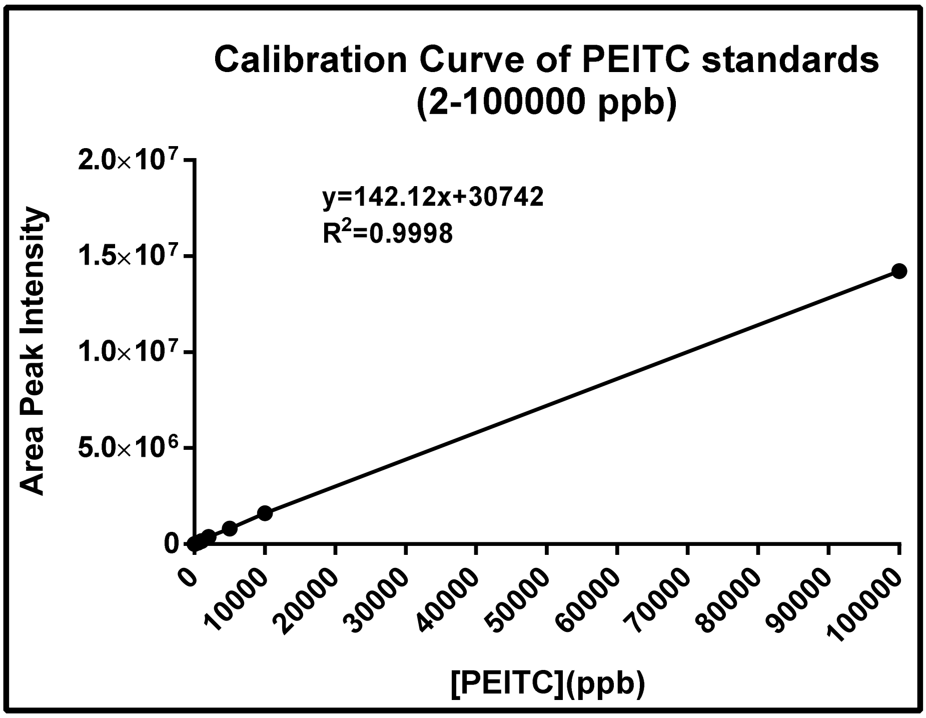

Figure S3: Calibration curve of various PEITC concentrations (2 ppb-100 ppm) used for the quantification of total PEITC obtained from both edible and non-edible watercress samples.

	Fractions	Run-1	Run-2	Run-3	Average	SD
**Edible Watercress sample**	Hexane	31964.2	32013.2	31862.01	31946.47	82.90
	Ethyl Acetate	31068.92	31241.96	32095.95	31468.94	459.92
	Chloroform	31086	31165.96	31881.99	31377.98	438.30
**Non-edible Watercress sample**	Hexane	30425.21	31964.21	31972.98	31454.02	891.08
	Ethyl Acetate	27785.11	31979.21	32464.32	30742.87	2572.96
	Chloroform	30735.11	30654.09	30837.06	30742.08	91.68

Table S2: Area peak intensity of three fractions from both edible and non-edible watercress samples. The quantification of total PEITC content was performed from three independed experiments (runs).
